# Tongaline

**Published:** 1884-08

**Authors:** 


TONGALINE.—We wish to call the attention of our readers to this new remedy for neuralgia and rheumatism. Having a case of neuralgia recently which did not improve under the ordinary treatment, we had Messrs. Bush & Co. order some Tongaline for us, which we gave to our patient. It acted admirably, relieving the pain before many doses had been taken. Since then we have had occasion to prescribe it several times, and with the same good results.

We believe Tongaline is destined to become "the" remedy for neuralgia, and the testimonials from noted physicians and surgeons surely tend to strengthen such a prediction. Try Tongaline and you will thank us for the suggestion.—Extract from the January ('84) number of the *Eastern Medical Journal*, Worcester, Mass.

